# Comparative Transcriptome Combined with Proteome Analyses Revealed Key Factors Involved in Alfalfa (*Medicago sativa*) Response to Waterlogging Stress

**DOI:** 10.3390/ijms20061359

**Published:** 2019-03-18

**Authors:** Ningbo Zeng, Zhijian Yang, Zhifei Zhang, Longxing Hu, Liang Chen

**Affiliations:** 1 Department of Pratacultural Sciences, College of Agriculture, Hunan Agricultural University, Changsha 410128, China; znb@hunau.edu.cn (N.Z.); youtikenn@163.com (Z.Y.); zhangzf@hunau.edu.cn (Z.Z.); 2 CAS Key Laboratory of Plant Germplasm Enhancement and Specialty Agriculture, Wuhan Botanical Garden, The Chinese Academy of Science, Wuhan 430074, China

**Keywords:** alfalfa, waterlogging, transcriptome, proteome, molecular mechanism

## Abstract

Alfalfa (*Medicago sativa*) is the most widely grown and most important forage crop in the world. However, alfalfa is susceptible to waterlogging stress, which is the major constraint for its cultivation area and crop production. So far, the molecular mechanism of alfalfa response to the waterlogging is largely unknown. Here, comparative transcriptome combined with proteomic analyses of two cultivars (M12, tolerant; M25, sensitive) of alfalfa showing contrasting tolerance to waterlogging were performed to understand the mechanism of alfalfa in response to waterlogging stress. Totally, 748 (581 up- and 167 down-regulated) genes were differentially expressed in leaves of waterlogging-stressed alfalfa compared with the control (M12_W vs. M12_CK), whereas 1193 (740 up- and 453 down-regulated) differentially abundant transcripts (DATs) were detected in the leaves of waterlogging-stressed plants in comparison with the control plants (M25_W vs. M25_CK). Furthermore, a total of 187 (122 up- and 65 down-regulated) and 190 (105 up- and 85 down-regulated) differentially abundant proteins (DAPs) were identified via isobaric tags for relative and absolute quantification (iTRAQ) method in M12_W vs. M12_CK and M25_W vs. M25_CK comparison, respectively. Compared dataset analysis of proteomics and transcriptomics revealed that 27 and eight genes displayed jointly up-regulated or down-regulated expression profiles at both mRNA and protein levels in M12_W vs. M12_CK comparison, whereas 30 and 27 genes were found to be co-up-regulated or co-down-regulated in M25_W vs. M25_CK comparison, respectively. The strongly enriched Kyoto Encyclopedia of Genes and Genomes (KEGG) pathways for co-up-regulated genes at mRNA and protein levels in M12_W vs. M12_CK comparison were ‘Amino sugar and nucleotide sugar metabolism’, ‘Arginine and proline metabolism’ and ‘Starch and sucrose metabolism’, whereas co-up-regulated protein-related pathways including ‘Arginine and proline metabolism’ and ‘Valine, leucine and isoleucine degradation’ were largely enriched in M25_W vs. M25_CK comparison. Importantly, the identified genes related to beta-amylase, Ethylene response Factor (ERF), Calcineurin B-like (CBL) interacting protein kinases (CIPKs), Glutathione peroxidase (GPX), and Glutathione-S-transferase (GST) may play key roles in conferring alfalfa tolerance to waterlogging stress. The present study may contribute to our understanding the molecular mechanism underlying the responses of alfalfa to waterlogging stress, and also provide important clues for further study and in-depth characterization of waterlogging-resistance breeding candidate genes in alfalfa.

## 1. Introduction

Alfalfa (*Medicago sativa*) is the most widely grown and most important forage crop in the world [1,2]. It is of great economic, environmental, and social values, such as providing food for livestock, enhancing soil fertility, N_2_ fixation, eliminating water contamination, and biofuels production [3,4]. However, the alfalfa is susceptible to waterlogging or water stress caused by excess rainfall and excessive irrigation, and this susceptibility is the major constraint for its cultivation area and crop production [2,5]. Therefore, to overcome the effects of waterlogging for alfalfa, it is essential to explore how alfalfa responds to the waterlogging.

As a serious abiotic stress, waterlogging threatens the growth and survival of plants because of oxygen deprivation [6]. Plants try to make metabolic or morphological changes, including glycolysis and formation of aerenchyma, to cope with the low oxygen conditions [7,8], and the process is always accompanied by specific alteration of transcription and translation. Genome-scale transcription studies have been performed in *Arabidopsis*, rice, maize, cotton, and other species in waterlogging stress [9,10,11]. For example, many transcription factors, belonging to AP2/ERF, WRKY, TGA, MYB, and bZIP families, were differentially expressed under waterlogging stress in kiwifruit [12]. Particularly, the ERF always accounted for the highest number of differentially expressed transcription factors under waterlogging conditions [13]. As reported, genes in response to waterlogging are mainly involved in ethylene synthesis, carbohydrate catabolism, lipid metabolism, glycolysis, ethanol fermentation, auxin-mediated processes, and reactive oxygen species (ROS) generation [14,15].

In addition, proteomic analysis has been widely used to detect differentially regulated proteins under waterlogging in maize, tomato, soybean, wheat [16,17,18,19,20]. Many anaerobically induced polypeptides (ANPs) which are essential for hypoxic tolerance have been identified by proteomics research in diverse plants [21]. Most of these ANPs are involved in the fermentation and glycolysis process, including sucrose synthase, glucose-6-phosphate isomerase, aldolase, and enolase [22,23]. Alam et al. discovered several novel waterlogging-responsive proteins that were not known previously as being waterlogging responsive except for the above well-known classical anaerobically induced proteins. The novel proteins were involved in several processes, i.e., signal transduction, programmed cell death, RNA processing, redox homeostasis, and metabolisms of energy [24]. 

Despite the molecular mechanism of the response to waterlogging have been reported in many studies, large differences in the transcriptional and translational regulation of above pathways are observed between species [25], and there have been relatively few studies of transcriptomic and proteomic changes in alfalfa until now. In this study, to understand the mechanism of the waterlogging response in alfalfa, the high throughput RNA-sequencing and iTRAQ analyses of two cultivars (M12 and M25) of alfalfa showing different tolerances to waterlogging stress were performed. Lists of candidate genes which may be related to waterlogging response in alfalfa were obtained by correlation analysis of the differentially abundant transcripts and proteins. This work will enhance understanding the mechanisms of waterlogging response in alfalfa, and may aid in stress resistance breeding of alfalfa. 

## 2. Results

### 2.1. Physiological Response to Waterlogging

**Figure 1:**
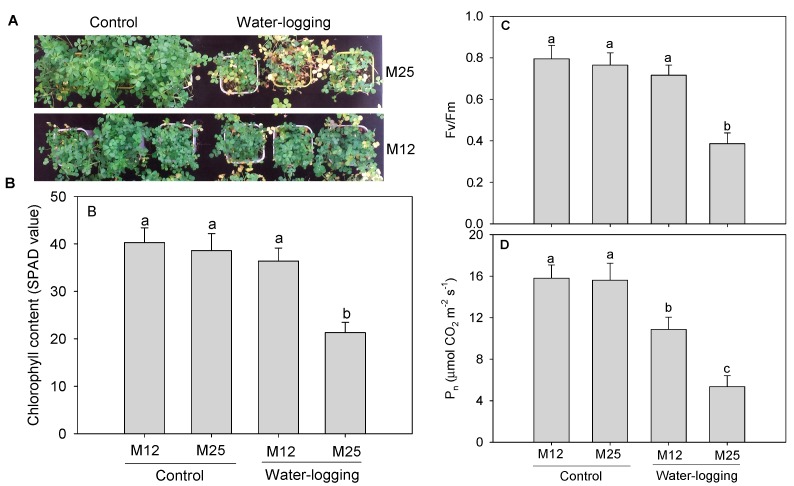
Effect of waterlogging on the phenotypic trait (**A**), leaf chlorophyll content (**B**), maximum quantum yield of photosystem II efficiency (Fv/Fm, (**C**) and net photosynthetic rate (P_n_) (**D**) in two alfalfa cultivars with contrasting waterlogging tolerance (M12: tolerant; M25, sensitive). Vertical bars on the top indicate standard deviation, and bars with the same letter indicate no significant difference at *p* < 0.05 for the comparison of different treatments (Duncan’s multiple range test).

Under well-watered control conditions, there was no significant difference between two cultivars (Figure 1A,B). When subjected to 12 days of waterlogging stress, the M25 showed a remarkable symptom of leaf senescence and chlorosis, as indicated by the decreased chlorophyll content (Figure 1B), whereas a slight reduction in chlorophyll content was observed in M12 after 12 days of waterlogging. Leaf Fv/Fm significantly declined for M25 after waterlogging, which reduced to 50% of the control level. For M12, however, Fv/Fm decreased only 10% when compared to the control plants (Figure 1C). Leaf P_n_ remarkably declined for M12 and M25 after waterlogging, which decreased by 31% and 66% when compared to the respective control plants (Figure 1D).

### 2.2. Transcriptome Sequencing and Assembly

To investigate the genes associated with waterlogging stress response, four cDNA libraries were constructed from total RNA extracted from leaves of alfalfa (M12 and M25) with or without waterlogging treatment. The libraries were then sequenced using the Illumina HiSeq™ 2000 platform. An overview of sequence assembly after illumina sequencing was shown in Table 1. A total of 180,507 transcripts were obtained from the clean reads with a mean length of 726 bp and length ranging from 201 to 15,720 bp (Table 2). Furthermore, 112,464 unigenes were obtained with an average length of 995 bp. The N50 and N90 for unigenes was 1448 and 456 bp, respectively.

### 2.3. Gene ontology (GO) and KOG Classification

**Figure 2:**
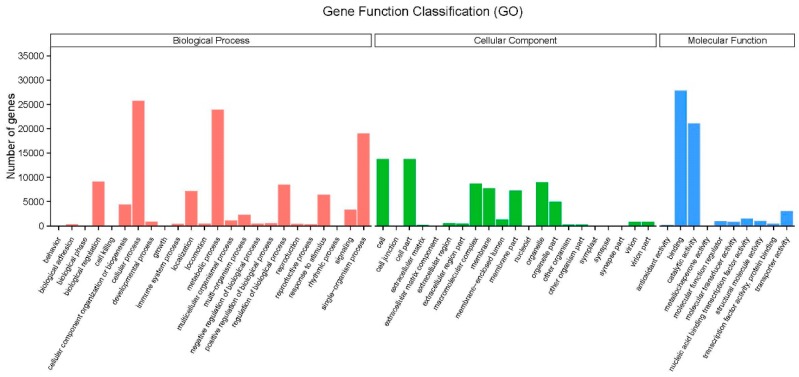
Histogram of gene ontology (GO) classification. The results are summarized in three main categories: biological process, cellular component, and molecular function.

After gene annotation, GO analysis was performed. A total of 46,339 genes were divided into three ontologies (Figure 2). For molecular function (MF) category, genes related to binding (27,870), ‘catalytic activity’ (21,044), ‘transporter activity’ (3018), and ‘nucleic acid binding transcription factor activity’ were highly represented. In terms of biological process (BP) category, it mainly comprised genes involved in ‘cellular process’ (25,688), ‘metabolic process’ (23,896), and ‘single-organism process’ (19,011). The highly represented GO Term (Lev2) was ‘cell’, ‘cell part’, and ‘organelle’ in the cellular component (CC) category.

**Figure 3:**
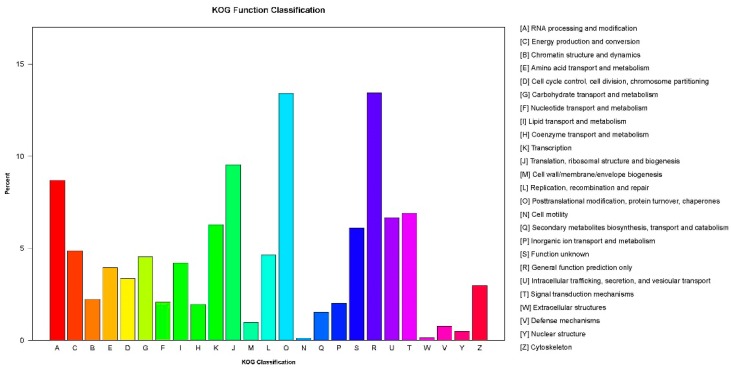
The euKaryotic Ortholog Groups (KOG) annotation of putative proteins. All 13,371 putative proteins assigned to KOG classification and classified into 25 molecular families.

There were 13,371 genes assigned to euKaryotic Ortholog Groups (KOG) classification and divided into 25 specific categories (Figure 3). The largest number of genes belonged to ‘general functional prediction only’ (1796) category, while only a few genes were divided into ‘Cell motility’ category.

### 2.4. Differentially Abundant Transcripts (DATs) Analysis

**Figure 4:**
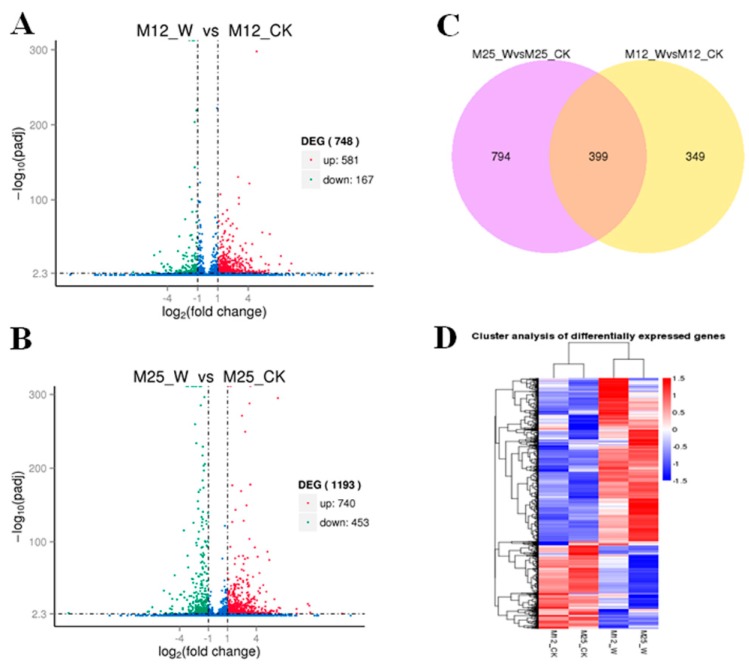
Volcano plots of differentially abundant transcripts in waterlogging-tolerant (**A**, M12) and waterlogging-sensitive (**B**, M25) plants after RNA-seq analysis. The *x*-axis represents the natural logarithm of fold change (Fc) and the *y*-axis represents log10 of the *p*-value of each transcript; (**C**) Differentially abundant transcripts showed in Venn diagram form; (**D**) hierarchical clustering analysis of waterlogging-induced changes in transcripts in leaves of alfalfa (M12CK indicates M12 under control condition; M12W indicates M12 under waterlogged condition; M25CK indicates M25 under control condition; M25W indicates M25 under waterlogged condition).

Totally, 748 (581 up- and 167 down-regulated) genes were differentially expressed in leaves of waterlogging-stressed alfalfa compared with control (M12_W vs. M12_CK) (Figure 4A, Supplementary File 1), whereas 1193 (740 up- and 453 down-regulated) DATs were detected in leaves of waterlogging-stressed plants in comparison with control plants (M25_W vs. M25_CK) (Figure 4B, Supplementary File 2).There were 399 overlapped DATs between M12_W vs. M12_CK and M25_W vs. M25_CK (Figure 4C). Further hierarchical clustering method was adopted to observe the overall expression pattern of DATs (Figure 4D). Most of the DATs showed remarkable differences in expression levels in waterlogging-treated conditions as compared to controls (Figure 4D).

### 2.5. Function Annotation of DATs Using the KEGG Database

The significantly enriched pathway terms were determined based on the *p*-Value and corrected *p*-Value according to the number of DATs in each term of the KEGG pathway in comparison with the background number. The top-four KEGG pathways associated with ‘Photosynthesis-antenna proteins’, ‘Arginine and proline metabolism’, ‘α-Linolenic acid metabolism’, and ‘Nitrogen metabolism’ were significantly enriched in the waterlogging-treated alfalfa versus the controls (M12_W vs. M12_CK) (*q* < 0.05). By contrast, the significantly enriched pathway terms in M25_W vs. M25_CK comparison were as follows: ‘Photosynthesis’, ‘Photosynthesis - antenna proteins’, ‘Carbon fixation in photosynthetic organisms’, ‘Nitrogen metabolism’, ‘Glyoxylate and dicarboxylate metabolism’, ‘Valine, leucine and isoleucine degradation’, ‘Arginine and proline metabolism’, ‘Porphyrin and chlorophyll metabolism’, ‘Fatty acid degradation’,‘Ubiquinone and other terpenoid-quinone biosynthesis’, and ‘Cysteine and methionine metabolism’ (Table 3).

### 2.6. Proteome-Wide Analysis of Differentially Abundant Proteins by Waterlogging Treatment

**Figure 5:**
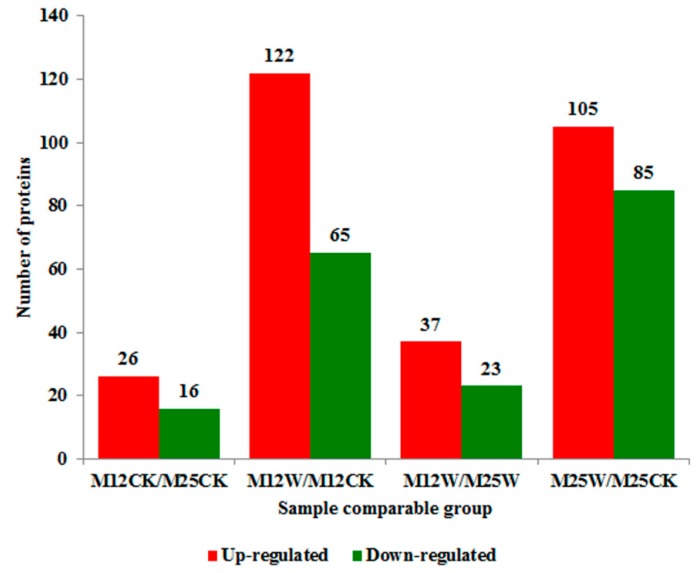
The number of differentially abundant proteins (fold change ≥ 1.3 or fold change ≤ 0.77 and *p*-value < 0.05) in two alfalfa cultivars (M12 and M25) under waterlogged and control conditions. M12CK indicate M12 under control condition; M12W indicate M12 under waterlogged condition; M25CK indicate M25 under control condition; M25W indicate M25 under waterlogged condition.

To further explore the differentially abundant proteins (DAPs) regulated by waterlogging, the iTRAQ method was applied. The mass spectrometry proteomics data have been deposited to the ProteomeXchange Consortium via the Proteomics Identifications (PRIDE) partner repository with the dataset identifier PXD013025. Totally, 3977 proteins were identified from alfalfa, among which 3436 proteins were quantified (Supplementary File 3). After setting the different fold changes >1.3 or <0.77 in one sample relative to the other sample as threshold, DAPs were obtained. A total of 187 (122 up- and 65 down-regulated) and 190 (105 up- and 85 down-regulated) DAPs were identified in M12_W vs. M12_CK and M25_W vs. M25_CK comparison, respectively (Figure 5, Supplementary File 3).

### 2.7. Gene Ontology (GO) Analysis of DAPs

During the M12_W vs. M12_CK comparison, ‘metabolic process’, ‘cellular process’, ‘single-organism process’, and ‘response to stimulus’ were the predominant components for ‘Biological Process’ category; while ‘cell’, ‘macromolecular complex’ and ‘organelle’ were highly represented in the ‘Cellular Component’ category. In the ‘Molecular Function’ category, proteins involved in ‘catalytic activity’, ‘binding’, ‘structural molecule activity’, and ‘antioxidant activity’ were the core components (Supplementary File 4). The similar results were also revealed in M25_W vs. M25_CK comparison (Supplementary File 5).

### 2.8. KEGG Analysis of DAPs

**Figure 6:**
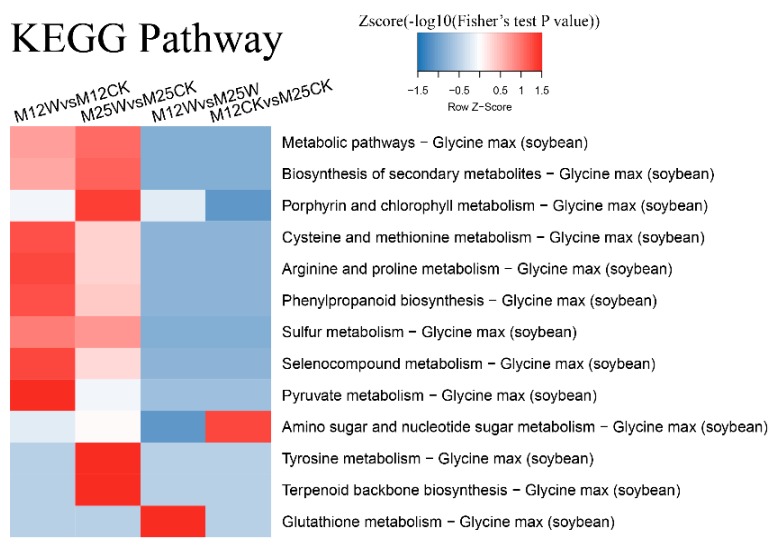
KEGG pathway analysis of differentially abundant proteins in two alfalfa cultivars (M12 and M25) under waterlogged and control conditions. M12CK indicates M12 under control condition; M12W indicates M12 under waterlogged condition; M25CK indicates M25 under control condition; M25W indicates M25 under waterlogged condition.

The pathways associated with ‘Cysteine and methionine metabolism’, ‘Arginine and proline metabolism’, ‘Phenylpropanoid biosynthesis’, ‘Seleno compound metabolism’, and ‘Pyruvate metabolism’ were predominantly enriched in M12_W vs. M12_CK comparison. Besides, ‘Biosynthesis of secondary metabolites’ and ‘Sulfur metabolism’ pathways were also moderately activated. By contrast, the highly enriched pathways were ‘Porphyrin and chlorophyll metabolism’, ‘Tyrosine metabolism’, ‘Terpenoid backbone biosynthesis’, and ‘Biosynthesis of secondary metabolites’, while ‘Sulfur metabolism’ was moderately enriched in M25_W vs. M25_CK comparison. Moreover, ‘Cysteine and methionine metabolism’, ‘Arginine and proline metabolism’, ‘Phenylpropanoid biosynthesis’, and ‘Seleno compound metabolism’ were also slightly enriched (Figure 6).

### 2.9. Transcriptomes and Proteomics Crosstalk Analysis

Compared dataset of proteomics and transcriptomics in M12_W vs. M12_CK comparison, 3853 proteins or transcripts were identified both in proteomics and transcriptomics research. Among which transcript and protein expression level of 3681 genes remained unchanged; 27 and eight genes displayed jointly up-regulated or down-regulated expression profiles at both mRNA and protein levels, respectively (Table 4). On the other hand, there were 3851 proteins or transcripts identified both in proteomics and transcriptomics research in M25_W vs. M25_CK comparison. Transcript or protein expression levels of 3475, 30, and 27 genes were found to be unchanged, co-up-regulated or co-down-regulated, respectively (Table 5). To confirm the transcript expression both at proteomic and transcriptomic levels, nine co-up-regulated or co-down-regulated target genes were selected to validate the RNA-seq and iTRAQ results by qPCR, and the results showed the same expression tendency as the RNA-seq and iTRAQ results (Supplementary File 6).

**Figure 7:**
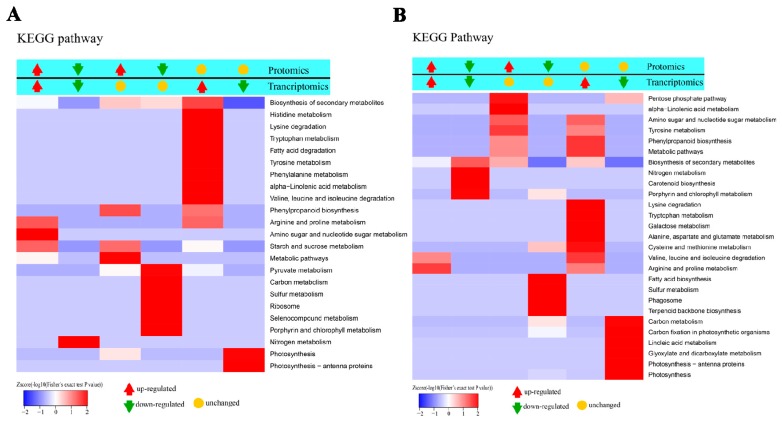
KEGG pathways for co-regulated genes at mRNA and protein levels in two alfalfa cultivars (**A**, M12W vs. M12CK; **B**, M25W vs. M25CK) under waterlogged and control conditions. M12CK indicates M12 under control condition; M12W indicates M12 under waterlogged condition; M25CK indicates M25 under control condition; M25W indicates M25 under waterlogged condition.

The strongly enriched KEGG pathways for co-up-regulated genes at mRNA and protein levels in M12_W vs. M12_CK comparison were ‘Amino sugar and nucleotide sugar metabolism’, ‘Arginine and proline metabolism’ and ‘Starch and sucrose metabolism (Figure 7A), whereas co-up-regulated protein-related pathways including ‘Arginine and proline metabolism’ and ‘Valine, leucine and isoleucine degradation’ were largely enriched in M25_W vs. M25_CK comparison (Figure 7B). As for waterlogging-down-regulated gene/protein-enriched pathways, ‘Nitrogen metabolism’ in ‘M12_W vs. M12_CK’ (Figure 7A) and ‘Nitrogen metabolism’, ‘Porphyrin and chlorophyll metabolism’, ‘Carotenoid biosynthesis’, and ‘Biosynthesis of secondary metabolites’ in ‘M25_W vs. M25_CK’ comparison were the predominant pathways (Figure 7B).

## 3. Discussion

Waterlogging restricts O_2_ diffusion and thereby inhibits aerobic respiration to plants. One of the adaptive strategies that plants survive periods of hypoxia (limited O_2_) or anoxia (no O_2_) is to generate energy through fermentative metabolism [26]. The fermentation, which is the main carbohydrate metabolism pathway during anaerobic condition, generates ATP and essential metabolites such as Ala, lactate, ethanol, and acetate in plants. During waterlogging stress, the energy metabolism of the plant is in a state of demand greater than supply. Moreover, the content of soluble sugar in plants is limited. Thus, the content of soluble sugar is vital for plants against waterlogging stress-induced injury. Studies have revealed that amylases play major roles in catalyzing carbohydrates conversion into soluble sugar [27]. In the present study, the expression of one gene (Cluster-1252.44338) encoding beta-amylase was induced 2.8-fold in waterlogging-resistant cultivar M12 after waterlogging treatment, while its expression was not significantly changed in waterlogging-sensitive cultivar M25. The enriched KEGG pathways consisting of co-up-regulated genes at both mRNA and protein levels were ‘Amino sugar and nucleotide sugar metabolism’, ‘Arginine and proline metabolism’, and ‘Starch and sucrose metabolism’ in M12_W vs. M12_CK comparison, and ‘Arginine and proline metabolism’ and ‘Valine, leucine and isoleucine degradation’ pathways in M25_W vs. M25_CK comparison. These results suggested that pathways related to carbohydrate and amino metabolism were activated when Alfalfa was exposed to waterlogging stress.

Plants adaptation to submergence stress includes two different opposing strategies, the quiescence strategy and the escape strategy. Plants temporarily stop shoot elongation to conserve energy as a quiescence strategy and resume growth when the water level is reduced. Conversely, as for escape strategy, plants keep their leaves above the water surface by stem elongation. The SUB1 locus on chromosome 9 containing a cluster of three group VII ethylene response factor (ERF) genes (SUB1A, SUB1B, and SUB1C), was identified to be involved in regulating the quiescence strategy via quantitative trait locus (QTL) mapping. The SUB1A-1 suppresses ethylene production, resulting in reduction of GA synthesis; the SUB1A-1 allele presence restricts undersurface shoot growth. Interestingly, two ERF family genes in deepwater rice named SNORKEL1 and SNORKEL2 (SK1/2), were revealed as positive regulators of internode elongation. In the present study, Ethylene-responsive transcription factor ERF110 (Cluster-1252.13837) was significantly induced by waterlogging treatment in both M12 and M25 cultivars, but the induction fold was higher in M25 (Log_2_-fold change 3.47) than that of M12 (Log_2_-fold change 2.29). Besides, ERF061 (Cluster-1252.45693) gene expression was up-regulated in M25 cultivar, but not in M12 cultivar. These results revealed that ERF genes play essential roles in alfalfa response to waterlogging. The differential expression levels of *ERF110* gene in waterlogging resistant cultivar M12 and waterlogging sensitive cultivar M25, and specifically expressed *ERF061* gene in M25 under waterlogging treatment condition may result in the different flood resistance ability. The roles of these *ERF* genes in waterlogging stress in alfalfa need to be further investigated.

Calcium signals are involved in plant responses to various stimuli, including abiotic- and biotic stresses, and regulate a wide range of physiological processes [28]. Ca^2+^ has also been proposed to regulate low O_2_ signaling in plants as the studies revealed that cytosolic Ca^2+^ concentration was transiently increased after flooding of maize roots or anoxic or hypoxic treatment of Arabidopsis [29,30]. Calcineurin B-like (CBL) proteins and their interacting protein kinases (CIPKs) transduce plant Ca^2+^ signaling through a complex network [31]. Lee et al. (2009) reported that the CIPK15-SnRK1A-MYBS1-mediated sugar-sensing pathway contributes to O_2_ deficiency tolerance during rice seed germination [32]. A very recent study has revealed that natural variation in *OsCBL10* promoter is involved in flooding stress response during seed germination among rice subspecies [33]. Here, the expressions of two *CIPK* genes (Cluster-1252.49669, Cluster-1252.47248) were obviously induced by waterlogging stress in waterlogging-resistant cultivar M12, whereas only one *CIPK* gene transcripts was accumulated by waterlogging treatment in waterlogging-sensitive cultivar M25 (Cluster-1252.43363). These results suggested that CIPK15--mediated calcium signals also play crucial roles in waterlogging stress in alfalfa.

Different abiotic stresses including waterlogging are known to cause the accumulation of reactive oxygen species (ROS) such as singlet oxygen, superoxide anion, hydrogen peroxide, and hydroxyl radicals which lead to membrane lipid peroxidation [34]. To minimize ROS induced injury for plant cells, plants adopt an antioxidant defense system, including non-enzymatic and enzymatic components to scavenge the excess ROS [35,36]. Among enzymatic antioxidants, Glutathione peroxidase (GPX) and Glutathione-S-transferase (GST) play important roles in protecting organisms from oxidative stress. In the present research, transcripts of five genes encoding GST and one gene encoding GPX were all obviously induced by waterlogging treatment in both M12 and M25 cultivars; however, the induction folds of these six genes were higher in M12 than those of M25 (Supplementary File 7). These results revealed that GST and GPX genes may play an important function in conferring M12 cultivar with enhanced tolerance to waterlogging.

## 4. Materials and Methods

### 4.1. Plant Materials and Waterlogging Treatment

Two alfalfa cultivars with contrasting waterlogging tolerance obtained from Beijing RYTWAY, M12 (tolerant) and M25 (sensitive), previously determined in our preliminary test, were used in this study. Equal amount of seeds for two cultivars were planted in pots with nursery substrate: river sand mix (2:1 *v*/*v*) in a greenhouse at Hunan Agricultural University under natural lighting in March 17th, 2017, with 18 pots per species. Plants were thinned to 15 seedlings in each pot two weeks after sowing. Four weeks’ old plants were moved to a growth chamber (Percival, Boone, Iowa, USA) with the following growth conditions: 25 °C/23 °C temperature (day/night), 14/10 h photoperiod, 500 µmol·m^−2^·s^−1^ light intensity, and 60–85% relative humidity. After one week of acclimation, plants were randomly assigned to “control” and “waterlogging” groups. For treatment, plants were subjected to waterlogging by immersing the plastic pots into water-filled plastic tubs by maintaining 1 cm water layer above soil surface, whereas the control pots were watered regularly to keep as 100% field soil water capacity. After 12 days’ treatment, samples were frozen by liquid nitrogen and were stored in −80 °C until use.

### 4.2. Leaf Chlorophyll Content, Maximum Quantum Yield of Photosystem II Efficiency (Fv/Fm), and Net Photosynthetic Rate (Pn) Determination

Leaf chlorophyll content was measured on the forth leaves from top by using a hand-held chlorophyll meter (SPAD-502, Spectrum Technologies Inc., Plainfield, IL, USA). Leaf maximum quantum yield of photosystem II efficiency (Fv/Fm) was evaluated by using a chlorophyll fluorometer (OS1-FL, Opti-Sciences, Hudson, NH, USA). Plants were adapted in darkness for 30 min and the then the measurements were made on intact leaves with the fluorometer.

Net photosynthetic rate (P_n_) was measured in the third leaves by using a gas analyzer (Li-6400, LICOR, Inc., Lincoln, NE, USA) with the controlled conditions (400 μmol·mol^−1^ CO_2_, 500 μmol·s^−1^ flow rate) and a LICOR 6400 LED external light source providing a photosynthetic photon flux density of 500 μmol·m^−2^·s^−1^.

### 4.3. RNA Preparation, Sequencing, and Data Analysis

Leaves total RNA was extracted using Trizol reagent (Invitrogen, Carlsbad, CA, USA) and purified using the RNeasy Plant Mini kit (Qiagen, Hilden, Germany) according to the Handbook. The quality and integrity of RNA was checked by Agilent Bioanalyzer 2100 system (Agilent Technologies, Palo Alto, CA, USA) and agarose gel electrophoresis.

A total amount of 1.5 µg RNA per sample was used as input material for the RNA sample preparations. Sequencing libraries were generated using NEBNext^®^ Ultra™ RNA Library Prep Kit for Illumina^®^ (NEB, Ipswich, MA, UK) following manufacturer’s recommendations and index codes were added to attribute sequences to each sample. Briefly, mRNA was purified from total RNA using poly-T oligo-attached magnetic beads. Fragmentation was carried out using divalent cations under elevated temperature in NEBNext First Strand Synthesis Reaction Buffer (5×). First strand cDNA was synthesized using random hexamer primer and M-MuLV Reverse Transcriptase (RNase H). Second strand cDNA synthesis was subsequently performed using DNA Polymerase I and RNase H. Remaining overhangs were converted into blunt ends via exonuclease/polymerase activities. After adenylation of 3′ ends of DNA fragments, NEBNext Adaptor with hairpin loop structure were ligated to prepare for hybridization. In order to select cDNA fragments of preferentially 150–200 bp in length, the library fragments were purified with AMPure XP system (Beckman Coulter, Beverly, MA, USA). Then 3 mm^3^ USER Enzyme (NEB) was used with size-selected, adaptor-ligated cDNA at 37 °C for 15 min followed by 5 min at 95 °C before PCR. Then PCR was performed with Phusion High-Fidelity DNA polymerase, Universal PCR primers and Index (X) Primer. At last, PCR products were purified (AMPure XP system) and library quality was assessed on the Agilent Bioanalyzer 2100 system. The clustering of the index-coded samples was performed on a cBot Cluster Generation System using TruSeq PE Cluster Kit v3-cBot-HS (Illumia, Santiago, CA, USA) according to the manufacturer’s instructions. After cluster generation, the library preparations were sequenced on an Illumina Hiseq platform and paired-end reads were generated. Raw data (raw reads) of fastq format were firstly processed through in-house perl scripts. In this step, clean data (clean reads) were obtained by removing reads containing adapter, reads containing ploy-*N* and low quality reads from raw data. At the same time, Q20, Q30, GC-content and sequence duplication level of the clean data were calculated. All the downstream analyses were based on clean data with high quality. Transcriptome assembly was accomplished using Trinity [37] with min_kmer_cov set to 2 by default and all other parameters set default.

Gene expression levels were estimated by RSEM [38] for each sample. Prior to differential gene expression analysis, for each sequenced library, the read counts were adjusted by edge R program package through one scaling normalized factor. Differential expression analysis of two samples was performed using the DEGseq [39] R package. *p*-value was adjusted using *q* value [40]. The *q*-value was set as the threshold for significantly differential expression.

Gene Ontology (GO) enrichment analysis of the differentially abundant transcripts (DATs) was implemented by the GOseq R packages based Wallenius non-central hyper-geometric distribution [41], which can adjust for gene length bias in DATs. KEGG [42] is a database resource for understanding high-level functions and utilities of the biological system, such as the cell, the organism and the ecosystem, from molecular-level information, especially large-scale molecular datasets generated by genome sequencing and other high-throughput experimental technologies (http://www.genome.jp/kegg/). We used KOBAS [43] software to test the statistical enrichment of differential expression genes in KEGG pathways.

### 4.4. Protein Extraction and Trypsin Digestion

The sample was ground using a mortar and a pestle in liquid nitrogen into cell powder and then transferred to a 5 cm^3^ centrifuge tube. After that, four volumes of lysis buffer (8 M urea, 1% Triton-100, 10 mM dithiothreitol, and 1% Protease Inhibitor Cocktail) was added to the cell powder, followed by sonication three times on ice using a high intensity ultrasonic processor (Ningbo Scientz Biotechnology Co., Ningbo, China). The remaining debris was removed by centrifugation at 20,000 g at 4 °C for 10 min. Finally, the protein was precipitated with cold 20% TCA for 2 h at −20 °C. After centrifugation at 12,000× *g* 4 °C for 10 min, the supernatant was discarded. The remaining precipitate was washed with cold acetone for three times. The protein was redissolved in 8 M urea and the protein concentration was determined with BCA kit according to the manufacturer’s instructions.

For digestion, the protein solution was reduced with 5 mM dithiothreitol for 30 min at 56 °C and alkylated with 11 mM iodoacetamide for 15 min at room temperature in darkness. The protein sample was then diluted by adding 100 mM TEAB to urea concentration less than 2 M. Finally, trypsin was added at 1:50 trypsin-to-protein mass ratio for the first digestion overnight and 1:100 trypsin-to-protein mass ratio for a second 4 h-digestion.

### 4.5. iTRAQ Labeling

After trypsin digestion, peptide was desalted by Strata X C18 SPE column (Phenomenex) and vacuum-dried. Peptide was reconstituted in 0.5 M TEAB and processed according to the manufacturer’s protocol for iTRAQ kit. Briefly, one unit of iTRAQ reagent were thawed and reconstituted in acetonitrile. The peptide mixtures were then incubated for 2 h at room temperature and pooled, desalted and dried by vacuum centrifugation. Then, pooled proteins were then labeled with iTRAQ reagents as follows M12CK: 113 and 114 (control), M12W: 115 and 116, M25CK: 117 and 118, and M25W: 119 and 121, and incubated at room temperature for 2 h. All labeled peptides were then combined before being dried in a vacuum concentrator (Eppendorf Concentrator 5301, Hamburg, Germany).

### 4.6. HPLC Fractionation and LC-MS/MS Analysis

The tryptic peptides were fractionated into fractions by high pH reverse-phase HPLC using Agilent 300Extend C18 column (5 μm particles, 4.6 mm ID, 250 mm length). Briefly, peptides were first separated with a gradient of 8% to 32% acetonitrile (pH 9.0) over 60 min into 60 fractions. Then, the peptides were combined into 18 fractions and dried by vacuum centrifuging. The tryptic peptides were dissolved in 0.1% formic acid (solvent A), directly loaded onto a home-made reversed-phase analytical column (15-cm length, 75 μm i.d.). The gradient was comprised of an increase from 6% to 23% solvent B (0.1% formic acid in 98% acetonitrile) over 26 min, 23% to 35% in 8 min and climbing to 80% in 3 min then holding at 80% for the last 3 min, all at a constant flow rate of 400 μm^3^/min on an EASY-nLC 1000 UPLC system (Thermo Fisher Scientific, Bremen, Germany).

The peptides were subjected to NSI source followed by tandem mass spectrometry (MS/MS) in Q Exactive™ Plus (Thermo Fisher Scientific) coupled online to the UPLC. The electrospray voltage applied was 2.0 kV. The *m*/*z* scan range was 350 to 1800 for full scan, and intact peptides were detected in the Orbitrap at a resolution of 70,000. Peptides were then selected for MS/MS using NCE setting as 28 and the fragments were detected in the Orbitrap at a resolution of 17,500. A data-dependent procedure that alternated between one MS scan followed by 20 MS/MS scans with 15.0 s dynamic exclusion. Automatic gain control (AGC) was set at 5 × 10^4^. Fixed first mass was set as 100 *m*/*z*.

### 4.7. Database Search

The resulting MS/MS data were processed using Maxquant search engine (v.1.5.2.8). Protein identification was performed using Sequest HT engine against the UniprotKB *Medicago truncatula* database (update to June 9th, 2016, including 70,050 protein sequences) [44]. Trypsin/P was specified as cleavage enzyme allowing up to 2 missing cleavages. The mass tolerance for precursor ions was set as 20 ppm in First search and 5 ppm in Main search, and the mass tolerance for fragment ions was set as 0.02 Da. Carbamidomethyl on Cys was specified as fixed modification and oxidation on Met was specified as variable modifications. FDR was adjusted to <1% and minimum score for peptides was set >40.

### 4.8. Validation of Differentially Expressed Unigenes and Proteins by Quantitative Real-Time PCR (qRT-PCR) Analysis

Total RNA extraction, cDNA synthesis, and qRT-PCRs were performed as Zhang et al. (2019) [45] described. Briefly, 0.1 g fresh tissues were used for total RNA extraction by using Trizol reagent (Invitrogen). RNA quality and integrity were checked by Nanodrop 2000 and 0.8% agarose gel. Then, the first strand cDNA were synthesized from 2 µg of total RNA using oligo(dT) 12–18 primer with the cDNA synthesis kit (Fermentas, Burlington, ON, Canada). Primer sequences were listed in Supplementary File 6. The real-time RT-PCR was conducted in ABI7500 with a final volume of 20 mm^3^. The PCR conditions were as follows: 40 cycles of 95 °C denaturation for 5 s, and 52–55 °C annealing and extension for 20 s. The relative expression level of genes for each sample was calculated relative to a calibrator using the DDCT method as described by Livak and Schmittgen (2001) [46].

### 4.9. Statistical Analysis

Statistical analysis was carried out following the ANOVA analysis of variance using SAS for Windows (SAS Institute, Cary, NC, USA). Comparisons between means were carried out using Duncan’s multiple range tests at a significance level of *p* < 0.05.

## 5. Conclusions

**Figure 8:**
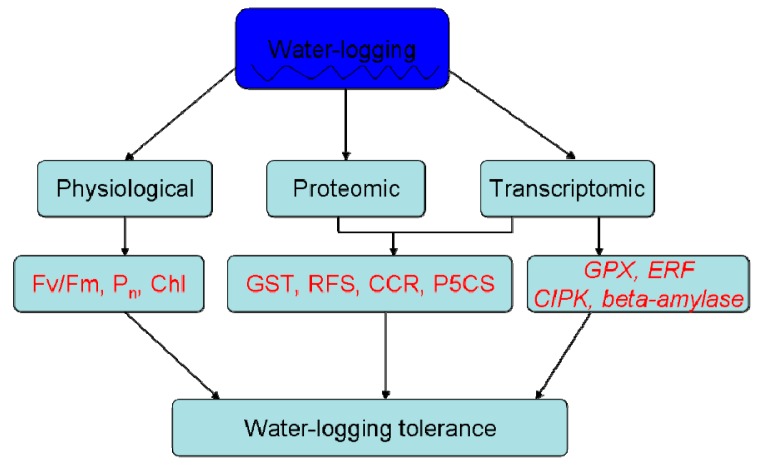
A schematic model for waterlogging stress responses in alfalfa plants.

The schematic figure summarized the alfalfa response to waterlogging stress with indicated difference between the two cultivars (Figure 8). Compared dataset analysis of proteomics and transcriptomics revealed that 27 and eight genes displayed jointly up-regulated or down-regulated expression profiles at both mRNA and protein levels in M12_W vs. M12_CK comparison, whereas 30 and 27 genes were found to be co-up-regulated or co-down-regulated in M25_W vs. M25_CK comparison, respectively. The strongly enriched Kyoto Encyclopedia of Genes and Genomes (KEGG) pathways for co-up-regulated genes at mRNA and protein levels in M12_W vs. M12_CK comparison were ‘Amino sugar and nucleotide sugar metabolism’, ‘Arginine and proline metabolism’, and ‘Starch and sucrose metabolism’, whereas co-up-regulated protein-related pathways including ‘Arginine and proline metabolism’ and ‘Valine, leucine and isoleucine degradation’ were largely enriched in M25_W vs. M25_CK comparison. Importantly, the identified genes related to beta-amylase, Ethylene response Factor (ERF), Calcineurin B-like (CBL) interacting protein kinases (CIPKs), Glutathione peroxidase (GPX), and Glutathione-S-transferase (GST) may play key roles in conferring alfalfa tolerance to waterlogging stress. The present study may contribute to our understanding the molecular mechanism underlying the responses of alfalfa to waterlogging stress, and also provide important clues for further study and in-depth characterization of waterlogging-resistance breeding candidate genes in alfalfa.

## Figures and Tables

**Table 1:** Summary of sequence assembly after illumina sequencing.

Sample	Raw Reads	Clean Reads	Clean Bases	Error (%)	Q 20 (%)	Q 30 (%)	GC (%)
M12_W	46788858	44893272	6.73G	0.02	96.00	90.30	41.61
M12_CK	53839716	51414672	7.71G	0.02	95.89	90.07	42.21
M25_W	50605680	48385890	7.26G	0.02	95.84	90.01	41.42
M25_CK	53716242	51239418	7.69G	0.02	95.70	89.65	42.09

**Table 2:** Length distribution of the transcripts and genes clustered from the de novo assembly.

Category	Transcripts	Genes
200–500 bp	105,740	39,418
500–1000 bp	36,445	34,759
1000–2000 bp	25,605	25,570
>2000 bp	12,717	12,717
Total	180,507	112,464
Min Length	201	201
Mean Length	726	995
Median Length	405	681
Max Length	15,720	15,720
N50	1196	1448
N90	283	456
Total Nucleotides	131,136,850	111,915,817

**Table 3:** Top 20 differentially abundant transcripts (DATs) enriched in Kyoto Encyclopedia of Genes and Genomes (KEGG) pathways.

Pathway Term	Rich Factor	*q* Value	Gene Number
M12_W vs. M12_CK			
Photosynthesis-antenna proteins	0.4	3.94 × 10^−8^	10
Arginine and proline metabolism	0.080882353	3.52 × 10^−3^	11
alpha-Linolenic acid metabolism	0.084745763	3.52 × 10^−3^	10
Nitrogen metabolism	0.116666667	4.51 × 10^−3^	7
Photosynthesis	0.067961165	6.70 × 10^−2^	7
Valine, leucine and isoleucine degradation	0.058333333	1.20 × 10^−1^	7
Carotenoid biosynthesis	0.065789474	1.67 × 10^−1^	5
Cysteine and methionine metabolism	0.044117647	1.67 × 10^−1^	9
Tropane, piperidine and pyridine alkaloid biosynthesis	0.076923077	1.67 × 10^−1^	4
Porphyrin and chlorophyll metabolism	0.057471264	1.77 × 10^−1^	5
Lysine degradation	0.056818182	1.77 × 10^−1^	5
Tryptophan metabolism	0.055555556	1.77 × 10^−1^	5
Isoquinoline alkaloid biosynthesis	0.085714286	1.77 × 10^−1^	3
Plant-pathogen interaction	0.031078611	1.77 × 10^−1^	17
Glutathione metabolism	0.040816327	1.77 × 10^−1^	8
Carbon fixation in photosynthetic organisms	0.047244094	1.77 × 10^−1^	6
Ascorbate and aldarate metabolism	0.052083333	1.77 × 10^−1^	5
Limonene and pinene degradation	0.069767442	2.22 × 10^−1^	3
Galactose metabolism	0.040540541	2.67 × 10^−1^	6
Glycerolipid metabolism	0.039735099	2.67 × 10^−1^	6
M25_W vs. M25_CK			
Photosynthesis	0.368932039	4.79 × 10^−20^	38
Photosynthesis—antenna proteins	0.68	3.55 × 10^−12^	17
Carbon fixation in photosynthetic organisms	0.204724409	5.17 × 10^−9^	26
Nitrogen metabolism	0.233333333	1.75 × 10^−5^	14
Glyoxylate and dicarboxylate metabolism	0.141732283	2.25 × 10^−4^	18
Valine, leucine and isoleucine degradation	0.141666667	3.28 × 10^−4^	17
Arginine and proline metabolism	0.132352941	3.68 × 10^−^	18
Porphyrin and chlorophyll metabolism	0.16091954	3.79 × 10^−4^	14
Fatty acid degradation	0.094339623	3.23 × 10^−2^	15
Ubiquinone and other terpenoid-quinone biosynthesis	0.11	3.54 × 10^−2^	11
Cysteine and methionine metabolism	0.083333333	4.67 × 10^−2^	17
Ascorbate and aldarate metabolism	0.104166667	6.19 × 10^−2^	10
Pentose phosphate pathway	0.093023256	6.19 × 10^−2^	12
Tryptophan metabolism	0.1	9.74 × 10^−2^	9
Carotenoid biosynthesis	0.105263158	1.0 × 10^−1^	8
Glycine, serine and threonine metabolism	0.077419355	1.63 × 10^−1^	12
Tyrosine metabolism	0.093023256	1.63 × 10^−1^	8
Alanine, aspartate and glutamate metabolism	0.085714286	1.73 × 10^−1^	9
Peroxisome	0.069444444	1.73 × 10^−1^	15
beta-Alanine metabolisma	0.075757576	2.33 × 10^−1^	10

**Table 4:** Differentially abundant transcripts both at proteomic and transcriptomic levels in M12 under waterlogging.

Protein Accession	Protein Description	MW (kDa)	M12W/M12CK Ratio	M12W/M12CK *p*-Value	Protein LOG2 M12W/M12CK	Regulation	Transcription Log2 M12_W/M12_CK	p adj	Regulation	Type
Cluster-1252.18690_orf1	Unknown	42.266	1.816	5.1953 × 10^−3^	0.860764203	Up	1.7749	9.0263 × 10^−7^	Up	Up-Up
Cluster-1252.20320_orf1	Probable mannitol dehydrogenase OS = Medicago sativa GN=CAD1 PE=1 SV=1	39.526	1.414	2.8745 × 10^−2^	0.49978212	Up	1.664	2.132 × 10^−4^	Up	Up-Up
Cluster-1252.24948_orf1	Acidic endochitinase OS=Cicer arietinum PE=2 SV=1	33.034	2.91	5.1816 × 10^−3^	1.541019153	Up	3.0386	4.4982 × 10^−18^	Up	Up-Up
Cluster-1252.25469_orf1	Probable glutathione S-transferase OS=Glycine max GN=HSP26-A PE=2 SV=1	26.335	2.141	1.02394 × 10^−3^	1.098284796	Up	2.0505	8.3503 × 10^−25^	Up	Up-Up
Cluster-1252.29574_orf1	Unknown	56.855	1.302	1.41228 × 10^−2^	0.380729449	Up	1.0417	3.2239 × 10^−4^	Up	Up-Up
Cluster-1252.33343_orf1	“Ornithine aminotransferase, mitochondrial OS=Arabidopsis thaliana GN=DELTA-OAT PE=1 SV=1”	51.439	1.503	4.2365 × 10^−8^	0.587845009	Up	1.4006	2.5266 × 10^−19^	Up	Up-Up
Cluster-1252.35376_orf1	Unknown	22.309	3.216	3.69926 × 10^−13^	1.685267407	Up	2.8743	1.7054 × 10^−62^	Up	Up-Up
Cluster-1252.36163_orf1	Expansin-like B1 OS=Arabidopsis thaliana GN=EXLB1 PE=2 SV=2	30.779	2.387	1.83814 × 10^−2^	1.255198566	Up	1.3227	1.0834 × 10^−4^	Up	Up-Up
Cluster-1252.38004_orf2	Protein C2-DOMAIN ABA-RELATED 9 OS=Arabidopsis thaliana GN=CAR9 PE=2 SV=1	21.642	1.534	7.6395 × 10^−5^	0.617298483	Up	1.4498	1.0054 × 10^−10^	Up	Up-Up
Cluster-1252.39652_orf1	18 kDa seed maturation protein OS=Glycine max GN=GMPM1 PE=2 SV=1	12.611	1.805	1.62154 × 10^−3^	0.851998837	Up	2.2232	4.4188 × 10^−12^	Up	Up-Up
Cluster-1252.41663_orf1	Stress-related protein OS=Phaseolus vulgaris GN=SRP PE=2 SV=1	146.66	1.7	3.5979 × 10^−5^	0.765534746	Up	1.1772	1.5419 × 10^−12^	Up	Up-Up
Cluster-1252.41945_orf1	Glucose-1-phosphate adenylyltransferase large subunit 1 (Fragment) OS=Solanum tuberosum GN=AGPS1 PE=2 SV=1	59.046	2.277	4.6658 × 10^−2^	1.187134291	Up	2.2953	1.4116 × 10^−4^	Up	Up-Up
Cluster-1252.42642_orf1	Unknown	16.163	3.21	9.99201 × 10^−16^	1.682573297	Up	1.6109	5.0453 × 10^−27^	Up	Up-Up
Cluster-1252.42960_orf1	Early nodulin-like protein 2 OS=Arabidopsis thaliana GN=At4g27520 PE=1 SV=1	103.53	1.698	1.90671 × 10^−5^	0.763836459	Up	1.0147	2.9677 × 10^−13^	Up	Up-Up
Cluster-1252.42962_orf1	Aldo-keto reductase family 4 member C9 OS=Arabidopsis thaliana GN=AKR4C9 PE=1 SV=1	36.025	1.447	5.56521 × 10^−12^	0.533064922	Up	1.5685	3.609 × 10^−25^	Up	Up-Up
Cluster-1252.43381_orf1	Thaumatin-like protein OS=Oryza sativa subsp. japonica GN=Os12g0628600 PE=1 SV=1	20.658	4.005	2.2805 × 10^−3^	2.001802243	Up	1.923	5.4445 × 10^−14^	Up	Up-Up
Cluster-1252.43664_orf1	Cinnamoyl-CoA reductase 1 OS=Arabidopsis thaliana GN=CCR1 PE=1 SV=1	34.902	2.775	1.0 × 10^−32^	1.472487771	Up	1.4878	7.6623 × 10^−82^	Up	Up-Up
Cluster-1252.43700_orf1	Vacuolar-processing enzyme OS=Vicia sativa PE=1 SV=1	148.25	1.998	2.2366 × 10^−3^	0.998556583	Up	1.237	4.6915 × 10^−108^	Up	Up-Up
Cluster-1252.44442_orf1	“Superoxide dismutase [Fe] 2, chloroplastic OS=Arabidopsis thaliana GN=FSD2 PE=1 SV=1”	36.823	1.725	3.0282 × 10^−10^	0.786596362	Up	1.0925	1.076 × 10^−3^	Up	Up-Up
Cluster-1252.45489_orf1	Unknown	87.568	1.436	2.8214 × 10^−4^	0.522055749	Up	1.1902	4.0089 × 10^−11^	Up	Up-Up
Cluster-1252.45907_orf1	Methylecgonone reductase OS=Erythroxylum coca PE=1 SV=1	35.219	1.928	1.2051 × 10^−7^	0.947105052	Up	1.071	1.6158 × 10^−5^	Up	Up-Up
Cluster-1252.48706_orf1	Delta-1-pyrroline-5-carboxylate synthase OS=Oryza sativa subsp. japonica GN=P5CS PE=2 SV=2	82.353	2.766	4.1957 × 10^−8^	1.467801156	Up	4.8411	2.0236 × 10^−298^	Up	Up-Up
Cluster-1252.50314_orf1	1-aminocyclopropane-1-carboxylate oxidase homolog 5 OS=Arabidopsis thaliana GN=2A6 PE=2 SV=2	47.587	2.108	7.0431 × 10^−11^	1.075874867	Up	2.6988	1.3474 × 10^−28^	Up	Up-Up
Cluster-1252.53143_orf1	“1,4-alpha-glucan-branching enzyme 1, chloroplastic/amyloplastic (Fragment) OS=Pisum sativum GN=SBEII PE=1 SV=1”	98.938	2.033	2.9402 × 10^−7^	1.023610215	Up	1.8785	8.7896 × 10^−8^	Up	Up-Up
Cluster-1252.59078_orf1	Pathogenesis-related protein PR-4B OS=Nicotiana tabacum PE=2 SV=1	19.406	2.258	3.0823 × 10^−2^	1.175045486	Up	2.4968	4.4984 × 10^−5^	Up	Up-Up
Cluster-1252.62468_orf1	Unknown	107.76	8.756	6.3273 × 10^−5^	3.130271955	Up	3.225	2.9758 × 10^−39^	Up	Up-Up
Cluster-1252.65950_orf1	Galactinol--sucrose galactosyltransferase OS=Pisum sativum GN=RFS PE=1 SV=1	90.573	1.834	6.6447 × 10^−10^	0.874993639	Up	2.8804	2.2919 × 10^−15^	Up	Up-Up
Cluster-1252.35865_orf1	Tubulin alpha chain OS=Prunus dulcis GN=TUBA PE=2 SV=1	54.36	0.648	2.3014 × 10^−3^	−0.625934282	Down	−1.5125	1.0164 × 10^−18^	Down	Down-Down
Cluster-1252.40636_orf1	1-aminocyclopropane-1-carboxylate oxidase OS=Prunus mume GN=ACO1 PE=2 SV=1	39.544	0.435	4.7576 × 10^−6^	−1.200912694	Down	−1.535	1.4095 × 10^−47^	Down	Down-Down
Cluster-1252.43303_orf1	“Ferredoxin--nitrite reductase, chloroplastic OS=Betula pendula GN=NIR1 PE=2 SV=1”	69.24	0.423	1.0 × 10^−32^	−1.241270432	Down	−2.8277	4.993 × 10^−52^	Down	Down-Down
Cluster-1252.43480_orf1	“Protochlorophyllide reductase, chloroplastic OS=Pisum sativum GN=3PCR PE=1 SV=1”	43.178	0.645	1.4187 × 10^−11^	−0.632628934	Down	−1.0364	1.1526 × 10^−50^	Down	Down-Down
Cluster-1252.43550_orf1	β-galactosidase 1 OS=Arabidopsis thaliana GN=BGAL1 PE=2 SV=1	93.798	0.506	2.2202 × 10^−5^	−0.98279071	Down	−1.5947	5.4211 × 10^−54^	Down	Down-Down
Cluster-1252.44318_orf1	Nitrate reductase [NADH] OS=Lotus japonicus GN=NIA PE=3 SV=1	102.62	0.623	1.33524 × 10^−3^	−0.682695932	Down	−2.0582	3.1915 × 10^−25^	Down	Down-Down
Cluster-1252.45309_orf1	Universal stress protein A-like protein OS=Arabidopsis thaliana GN=At3g01520 PE=1 SV=2	18.198	0.552	2.9101 × 10^−2^	−0.857259828	Down	−1.2288	1.3773 × 10^−29^	Down	Down-Down
Cluster-1252.48945_orf1	Unknown	40.668	0.512	2.36871 × 10^−11^	−0.965784285	Down	−2.4873	1.4274 × 10^−10^	Down	Down-Down

**Table 5:** Differentially abundant transcripts both at proteomic and transcriptomic levels in M25 under waterlogging.

Protein Accession	Protein Description	MW (kDa)	M25W/M25CK Ratio	M25W/M25CK *p*-Value	Protein LOG2 M25W/M25CK	Regulation	Transcription LOG2 M25W/M25CK	*p* adj	Regulation	Type
Cluster-1252.18690_orf1	Unknown	42.266	1.345	4.7545 × 10^−2^	0.427606173	Up	2.7094	1.029 × 10^−14^	Up	Up-Up
Cluster-1252.25469_orf1	Probable glutathione *S*-transferase OS=Glycine max GN=HSP26-A PE=2 SV=1	26.335	2.541	1.12492 × 10^−3^	1.345396375	Up	1.7162	7.0349 × 10^−27^	Up	Up-Up
Cluster-1252.27678_orf1	Cinnamoyl-CoA reductase 2 OS=Arabidopsis thaliana GN=CCR2 PE=1 SV=1	37.481	1.36	3.5464 × 10^−3^	0.443606651	Up	3.4894	1.0739 × 10^−7^	Up	Up-Up
Cluster-1252.32933_orf1	Probable cinnamyl alcohol dehydrogenase OS=Medicago sativa GN=CAD2 PE=1 SV=1	40.598	1.336	3.82 × 10^−4^	0.417920008	Up	1.0817	2.6648 × 10^−7^	Up	Up-Up
Cluster-1252.33343_orf1	“Ornithine aminotransferase, mitochondrial OS=Arabidopsis thaliana GN=DELTA-OAT PE=1 SV=1”	51.439	1.813	3.3792 × 10^−8^	0.858378925	Up	2.6159	1.0261 × 10^−81^	Up	Up-Up
Cluster-1252.35478_orf1	Unknown	46.282	1.401	1.0601 × 10^−9^	0.486456956	Up	1.59	1.3747 × 10^−13^	Up	Up-Up
Cluster-1252.36163_orf1	Expansin-like B1 OS=Arabidopsis thaliana GN=EXLB1 PE=2 SV=2	30.779	2.054	1.2356 × 10^−2^	1.038436182	Up	2.62	1.3061 × 10^−10^	Up	Up-Up
Cluster-1252.36198_orf1	Cytochrome b5 OS=Brassica oleracea var. botrytis GN=CYB5 PE=1 SV=1	17.483	1.312	1.59783 × 10^−4^	0.39176772	Up	1.1567	2.1863 × 10^−10^	Up	Up-Up
Cluster-1252.38004_orf2	Protein C2-DOMAIN ABA-RELATED 9 OS=Arabidopsis thaliana GN=CAR9 PE=2 SV=1	21.642	1.651	6.0141 × 10^−5^	0.72334012	Up	1.2091	4.96 × 10^−7^	Up	Up-Up
Cluster-1252.39652_orf1	18 kDa seed maturation protein OS=Glycine max GN=GMPM1 PE=2 SV=1	12.611	2.227	4.7834 × 10^−3^	1.155101558	Up	1.393	1.1811 × 10^−4^	Up	Up-Up
Cluster-1252.42092_orf1	Desiccation protectant protein Lea14 homolog OS=Glycine max PE=2 SV=1	51.585	1.35	1.00421 × 10^−3^	0.432959407	Up	1.8427	4.6211 × 10^−8^	Up	Up-Up
Cluster-1252.42169_orf1	4-hydroxyphenylpyruvate dioxygenase OS=Arabidopsis thaliana GN=HPD PE=1 SV=2	59.596	1.838	3.7081 × 10^−5^	0.878136767	Up	2.1711	3.1841 × 10^−81^	Up	Up-Up
Cluster-1252.42520_orf1	ABC transporter C family member 4 OS=Arabidopsis thaliana GN=ABCC4 PE=2 SV=2	194.56	1.426	1.39367 × 10^−2^	0.511973982	Up	1.1124	7.8224 × 10^−50^	Up	Up-Up
Cluster-1252.42642_orf1	Unknown	16.163	2.542	1.56475 × 10^−12^	1.34596403	Up	2.1592	1.2647 × 10^−37^	Up	Up-Up
Cluster-1252.42962_orf1	Aldo-keto reductase family 4 member C9 OS=Arabidopsis thaliana GN=AKR4C9 PE=1 SV=1	36.025	1.711	1.11022 × 10^−16^	0.77483976	Up	1.6703	1.2377 × 10^−41^	Up	Up-Up
Cluster-1252.43664_orf1	Cinnamoyl-CoA reductase 1 OS=Arabidopsis thaliana GN=CCR1 PE=1 SV=1	34.902	2.885	1.0 × 10^−32^	1.528571319	Up	1.0253	4.2827 × 10^−31^	Up	Up-Up
Cluster-1252.43700_orf1	Vacuolar-processing enzyme OS=Vicia sativa PE=1 SV=1	148.25	2.293	7.1779 × 10^−4^	1.197236355	Up	1.497	1.8971 × 10^−177^	Up	Up-Up
Cluster-1252.43923_orf1	“Crocetin glucosyltransferase, chloroplastic OS=Gardenia jasminoides GN=UGT75L6 PE=1 SV=1”	54.447	1.499	1.80426 × 10^−4^	0.584000383	Up	1.3321	1.6138 × 10^−5^	Up	Up-Up
Cluster-1252.44444_orf1	Malonate-CoA ligase OS=Arabidopsis thaliana GN=AAE13 PE=1 SV=1	69.952	1.312	1.37048 × 10^−4^	0.39176772	Up	1.9792	1.2674 × 10^−22^	Up	Up-Up
Cluster-1252.45456_orf1	Copper transport protein CCH OS=Arabidopsis thaliana GN=CCH PE=1 SV=1	15.409	2.06	2.3795 × 10^−2^	1.042644337	Up	1.0154	4.6715 × 10^−3^	Up	Up-Up
Cluster-1252.45489_orf1	Unknown	87.568	1.487	9.5623 × 10^−4^	0.572404647	Up	1.0995	7.6879 × 10^−12^	Up	Up-Up
Cluster-1252.46922_orf1	Aldehyde dehydrogenase family 7 member A1 OS=Pisum sativum PE=1 SV=3	54.83	1.407	4.1199 × 10^−7^	0.492622329	Up	2.4112	1.3416 × 10^−169^	Up	Up-Up
Cluster-1252.47893_orf1	Unknown	42.991	1.503	7.1367 × 10^−3^	0.587845009	Up	2.4434	1.0982 × 10^−38^	Up	Up-Up
Cluster-1252.48706_orf1	Delta-1-pyrroline-5-carboxylate synthase OS=Oryza sativa subsp. japonica GN=P5CS PE=2 SV=2	82.353	2.771	2.0696 × 10^−7^	1.470406711	Up	6.2581	7.2562 × 10^−296^	Up	Up-Up
Cluster-1252.49757_orf2	Unknown	28.735	1.329	1.89241 × 10^−2^	0.410341105	Up	1.6066	2.6525 × 10^−10^	Up	Up-Up
Cluster-1252.49800_orf1	β-galactosidase 8 OS=Arabidopsis thaliana GN=BGAL8 PE=2 SV=2	65.645	1.349	1.14055 × 10^−3^	0.431890348	Up	4.143	1.8906 × 10^−80^	Up	Up-Up
Cluster-1252.50314_orf1	1-aminocyclopropane-1-carboxylate oxidase homolog 5 OS=Arabidopsis thaliana GN=2A6 PE=2 SV=2	47.587	2.728	1.66533 × 10^−15^	1.447843644	Up	3.2213	3.1324 × 10^−40^	Up	Up-Up
Cluster-1252.53143_orf1	“1,4-α-glucan-branching enzyme 1, chloroplastic/amyloplastic (Fragment) OS=Pisum sativum GN=SBEII PE=1 SV=1”	98.938	1.681	3.2469 × 10^−6^	0.749319725	Up	1.6874	7.8729 × 10^−7^	Up	Up-Up
Cluster-1252.62468_orf1	Unknown	107.76	6.211	2.2964 × 10^−5^	2.634825568	Up	4.7334	5.8563 × 10^−42^	Up	Up-Up
Cluster-1252.65950_orf1	Galactinol-sucrose galactosyltransferase OS=Pisum sativum GN=RFS PE=1 SV=1	90.573	1.73	1.9153 × 10^−10^	0.790772038	Up	2.7024	3.5766 × 10^−5^	Up	Up-Up
Cluster-1252.38137_orf1	“Superoxide dismutase [Cu-Zn], chloroplastic OS=Medicago sativa GN=SODCP PE=2 SV=1”	24.007	0.76	3.2114 × 10^−4^	−0.395928676	Down	−3.946	5.8488 × 10^−29^	Down	Down-Down
Cluster-1252.40018_orf1	“CBS domain-containing protein CBSX3, mitochondrial OS=Arabidopsis thaliana GN=CBSX3 PE=1 SV=1”	26.605	0.752	1.1475 × 10^−11^	−0.411195433	Down	−1.274	3.2125 × 10^−18^	Down	Down-Down
Cluster-1252.40357_orf1	Unknown	23.476	0.658	9.9921 × 10^−4^	−0.603840511	Down	−1.2781	3.4128 × 10^−4^	Down	Down-Down
Cluster-1252.40636_orf1	1-aminocyclopropane-1-carboxylate oxidase OS=Prunus mume GN=ACO1 PE=2 SV=1	39.544	0.544	3.9251 × 10^−5^	−0.878321443	Down	−1.0541	5.7398 × 10^−15^	Down	Down-Down
Cluster-1252.42669_orf1	“50S ribosomal protein L19, chloroplastic OS=Spinacia oleracea GN=RPL19 PE=1 SV=2”	27.445	0.737	1.39429 × 10^−2^	−0.440263476	Down	−1.1371	9.5976 × 10^−9^	Down	Down-Down
Cluster-1252.43155_orf1	“30S ribosomal protein S17, chloroplastic (Fragment) OS=Pisum sativum GN=RPS17 PE=2 SV=1”	19.162	0.764	4.0527 × 10^−5^	−0.388355457	Down	−1.3192	2.386 × 10^−7^	Down	Down-Down
Cluster-1252.43183_orf1	Protein TSS OS=Arabidopsis thaliana GN=TSS PE=1 SV=1	180.86	0.718	4.32987 × 10^−15^	−0.477944251	Down	−1.2443	3.8189 × 10^−29^	Down	Down-Down
Cluster-1252.43187_orf1	“Thiamine thiazole synthase 2, chloroplastic OS=Vitis vinifera GN=THI1-2 PE=3 SV=1”	41.382	0.63	1.0979 × 10^−7^	−0.666576266	Down	−1.5012	3.85 × 10^−204^	Down	Down-Down
Cluster-1252.43232_orf1	“Probable carotenoid cleavage dioxygenase 4, chloroplastic OS=Arabidopsis thaliana GN=CCD4 PE=1 SV=1”	40.321	0.705	5.9396 × 10^−8^	−0.504304837	Down	−1.5008	4.5918 × 10^−20^	Down	Down-Down
Cluster-1252.43303_orf1	“Ferredoxin--nitrite reductase, chloroplastic OS=Betula pendula GN=NIR1 PE=2 SV=1”	69.24	0.482	1.0 × 10^−32^	−1.052894948	Down	−1.6604	1.2831 × 10^−26^	Down	Down-Down
Cluster-1252.43381_orf1	Thaumatin-like protein OS=Oryza sativa subsp. japonica GN=Os12g0628600 PE=1 SV=1	20.658	0.707	3.3377 × 10^−2^	−0.50021788	Down	−2.7087	1.6235 × 10^−50^	Down	Down-Down
Cluster-1252.43480_orf1	“Protochlorophyllide reductase, chloroplastic OS=Pisum sativum GN=3PCR PE=1 SV=1”	43.178	0.503	4.6423 × 10^−9^	−0.991369695	Down	−1.7186	7.5419 × 10^−93^	Down	Down-Down
Cluster-1252.43489_orf1	“Ketol-acid reductoisomerase, chloroplastic OS=Pisum sativum GN=PGAAIR PE=2 SV=1”	66.112	0.679	1.0 × 10^−32^	−0.55851652	Down	−1.0004	3.72 × 10^−8^	Down	Down-Down
Cluster-1252.43517_orf1	“Magnesium-protoporphyrin IX monomethyl ester [oxidative] cyclase, chloroplastic OS=Euphorbia esula GN=CRD1 PE=3 SV=1”	48.801	0.633	1.9456 × 10^−10^	−0.659722595	Down	−1.5422	1.5614 × 10^−85^	Down	Down-Down
Cluster-1252.43550_orf1	β-galactosidase 1 OS=Arabidopsis thaliana GN=BGAL1 PE=2 SV=1	93.798	0.442	2.3143 × 10^−5^	−1.177881725	Down	−1.9732	1.0767 × 10^−53^	Down	Down-Down
Cluster-1252.43873_orf1	“Light-harvesting complex-like protein 3 isotype 1, chloroplastic OS=Arabidopsis thaliana GN=LIL3.1 PE=1 SV=1”	29.313	0.761	1.4038 × 10^−6^	−0.394031641	Down	−1.6761	3.8258 × 10^−18^	Down	Down-Down
Cluster-1252.43934_orf1	“Probable carotenoid cleavage dioxygenase 4, chloroplastic OS=Arabidopsis thaliana GN=CCD4 PE=1 SV=1”	27.699	0.746	1.28296 × 10^−3^	−0.422752464	Down	−1.6188	2.6379 × 10^−10^	Down	Down-Down
Cluster-1252.44318_orf1	Nitrate reductase [NADH] OS=Lotus japonicus GN=NIA PE=3 SV=1	102.62	0.573	2.1975 × 10^−4^	−0.803392956	Down	−1.7715	2.6347 × 10^−42^	Down	Down-Down
Cluster-1252.45201_orf2	“Magnesium-chelatase subunit ChlI, chloroplastic OS=Glycine max GN=CHLI PE=2 SV=1”	47.591	0.681	1.8359 × 10^−11^	−0.554273297	Down	−1.8322	5.4646 × 10^−38^	Down	Down-Down
Cluster-1252.45508_orf1	“Porphobilinogen deaminase, chloroplastic OS=Pisum sativum GN=HEMC PE=1 SV=1”	45.052	0.665	1.0314 × 10^−12^	−0.588573754	Down	−1.0198	2.5422 × 10^−3^	Down	Down-Down
Cluster-1252.46447_orf1	“Magnesium protoporphyrin IX methyltransferase, chloroplastic OS=Arabidopsis thaliana GN=CHLM PE=1 SV=1”	39.446	0.724	2.9589 × 10^−4^	−0.465938398	Down	−3.5109	3.9122 × 10^−25^	Down	Down-Down
Cluster-1252.47724_orf1	Stem 28 kDa glycoprotein OS=Glycine max GN=VSPA PE=2 SV=1	30.865	0.753	1.58697 × 10^−5^	−0.40927823	Down	−1.5366	4.9784 × 10^−7^	Down	Down-Down
Cluster-1252.48703_orf1	“Glutamyl-tRNA reductase 1, chloroplastic OS=Cucumis sativus GN=HEMA1 PE=2 SV=1”	59.248	0.541	5.1604 × 10^−3^	−0.886299501	Down	−1.1181	2.0974 × 10^−5^	Down	Down-Down
Cluster-1252.48945_orf1	Unknown	40.668	0.611	7.1715 × 10^−11^	−0.710755715	Down	−3.236	6.0036 × 10^−22^	Down	Down-Down
Cluster-1252.49560_orf1	“Probable plastid-lipid-associated protein 8, chloroplastic OS=Arabidopsis thaliana GN=PAP8 PE=1 SV=1”	168.8	0.755	3.6428 × 10^−4^	−0.40545145	Down	−1.072	1.7811 × 10^−6^	Down	Down-Down
Cluster-1252.51383_orf1	“50S ribosomal protein L29, chloroplastic OS=Arabidopsis thaliana GN=RPL29 PE=1 SV=1”	20	0.756	1.67585 × 10^−3^	−0.40354186	Down	−1.6621	2.8565 × 10^−8^	Down	Down-Down
Cluster-1252.59847_orf1	ATP sulfurylase 2 OS=Arabidopsis thaliana GN=APS2 PE=1 SV=1	57.663	0.633	1.8719 × 10^−7^	−0.659722595	Down	−1.5753	1.7315 × 10^−3^	Down	Down-Down

## Supplementary Materials

Supplementary materials can be found at https://www.mdpi.com/1422-0067/20/6/1359/s1.

## References

[1] Bagavathiannan M.V., Van Acker R.C. (2009). The biology and ecology of feral alfalfa (*Medicago sativa* L.) and its implications for novel trait confinement in North America. Crit. Rev. Plant Sci..

[2] Barta A., Sulc R. (2002). Interaction between waterlogging injury and irradiance level in alfalfa. Crop Sci..

[3] Breazeale D., Neufeld J., Myer G., Davison J. (2000). Feasibility of subsurface drip irrigation for alfalfa. J. ASFMRA.

[4] Samac D.A., Jung H., Lamb J. (2006). Development of alfalfa (*Medicago sativa* L.) as a feedstock for production of ethanol and other bioproducts. Chem. Ind.-N. Y.-Marcel Dekk..

[5] Humphries A.W., Auricht G. (2001). Breeding lucerne for Australias southern dryland cropping environments. Aust. J. Agric. Res..

[6] Voesenek L.A., Bailey-Serres J. (2015). Flood adaptive traits and processes: An overview. New Phytol..

[7] Dat J.F., Capelli N., Folzer H., Bourgeade P., Badot P.M. (2004). Sensing and signalling during plant flooding. Plant Physiol. Biochem..

[8] Drew M.C. (1997). Oxygen deficiency and root metabolism: Injury and acclimation under hypoxia and anoxia. Ann. Rev. Plant Biol..

[9] Christianson J.A., Llewellyn D.J., Dennis E.S., Wilson I.W. (2009). Global gene expression responses to waterlogging in roots and leaves of cotton (*Gossypium hirsutum* L.). Plant Cell Physiol..

[10] Christianson J.A., Llewellyn D.J., Dennis E.S., Wilson I.W. (2010). Comparisons of early transcriptome responses to low-oxygen environments in three dicotyledonous plant species. Plant Signal. Behav..

[11] Nanjo Y., Maruyama K., Yasue H., Yamaguchi-Shinozaki K., Shinozaki K., Komatsu S. (2011). Transcriptional responses to flooding stress in roots including hypocotyl of soybean seedlings. Plant Mol. Biol..

[12] Zhang J.Y., Huang S.N., Mo Z.H., Xuan J.P., Jia X.D., Wang G., Guo Z.R. (2015). De novo transcriptome sequencing and comparative analysis of differentially expressed genes in kiwifruit under waterlogging stress. Mol. Breed..

[13] Chen W., Yao Q., Patil G.B., Agarwal G., Deshmukh R.K., Lin L., Wang B., Wang Y., Prince S.J., Song L. (2016). Identification and comparative analysis of differential gene expression in soybean leaf tissue under drought and flooding stress revealed by RNA-Seq. Front. Plant Sci..

[14] Klok E.J., Wilson I.W., Wilson D., Chapman S.C., Ewing R.M., Somerville S.C., Peacock W.J., Dolferus R., Dennis E.S. (2002). Expression profile analysis of the low-oxygen response in Arabidopsis root cultures. Plant Cell.

[15] Liu F., VanToai T., Moy L.P., Bock G., Linford L.D., Quackenbush J. (2005). Global transcription profiling reveals comprehensive insights into hypoxic response in Arabidopsis. Plant Physiol..

[16] Ahsan N., Lee D.G., Lee S.H., Kang K.Y., Bahk J.D., Choi M.S., Lee I.J., Renaut J., Lee B.H. (2007). A comparative proteomic analysis of tomato leaves in response to waterlogging stress. Physiol. Plant..

[17] Komatsu S., Kamal A.H., Hossain Z. (2014). Wheat proteomics: Proteome modulation and abiotic stress acclimation. Front. Plant Sci..

[18] Komatsu S., Shirasaka N., Sakata K. (2013). ‘Omics’ techniques for identifying flooding–response mechanisms in soybean. J. Proteom..

[19] Wang X., Huang M., Zhou Q., Cai J., Dai T., Cao W., Jiang D. (2016). Physiological and proteomic mechanisms of waterlogging priming improves tolerance to waterlogging stress in wheat (*Triticum aestivum* L.). Environ. Exp. Bot..

[20] Yu F., Han X., Geng C., Zhao Y., Zhang Z., Qiu F. (2015). Comparative proteomic analysis revealing the complex network associated with waterlogging stress in maize (*Zea mays* L.) seedling root cells. Proteomics.

[21] Mujer C.V., Rumpho M.E., Lin J.J., Kennedy R.A. (1993). Constitutive and inducible aerobic and anaerobic stress proteins in the Echinochloa complex and rice. Plant Physiol..

[22] Sachs M.M., Freeling M., Okimoto R. (1980). The anaerobic proteins of maize. Cell.

[23] Zhang Z., Zhang D., Zheng Y. (2009). Transcriptional and post-transcriptional regulation of gene expression in submerged root cells of maize. Plant Signal. Behav..

[24] Alam I., Lee D.G., Kim K.H., Park C.H., Sharmin S.A., Lee H., Oh K.W., Yun B.W., Lee B.H. (2010). Proteome analysis of soybean roots under waterlogging stress at an early vegetative stage. J. Biosci..

[25] Narsai R., Rocha M., Geigenberger P., Whelan J., van Dongen J.T. (2011). Comparative analysis between plant species of transcriptional and metabolic responses to hypoxia. New Phytol..

[26] Das A., Uchimiya H. (2002). Oxygen stress and adaptation of a semi-aquatic plant: Rice (*Oryza sativa*). J. Plant Res..

[27] Guglielminetti L., Perata P., Alpi A. (1995). Effect of anoxia on carbohydrate metabolism in rice seedlings. Plant Physiol..

[28] Harper J.F. (2001). Dissecting calcium oscillators in plant cells. Trends Plant Sci..

[29] Sedbrook J.C., Kronebusch P.J., Borisy G.G., Trewavas A.J., Masson P.H. (1996). Transgenic AEQUORIN reveals organ-specific cytosolic Ca^2+^ responses to anoxia in Arabidopsis thaliana seedlings. Plant Physiol..

[30] Subbaiah C.C., Zhang J., Sachs M.M. (1994). Involvement of intracellular calcium in Anaerobic gene expression and survival of maize seedlings. Plant Physiol..

[31] Batistic O., Kudla J. (2004). Integration and channeling of calcium signaling through the CBL calcium sensor/CIPK protein kinase network. Planta.

[32] Lee K.W., Chen P.W., Lu C.A., Chen S., Ho T.H.D., Yu S.M. (2009). Coordinated responses to oxygen and sugar deficiency allow rice seedlings to tolerate flooding. Sci. Signal..

[33] Ye N.H., Wang F.Z., Shi L., Chen M.X., Cao Y.Y., Zhu F.Y., Wu Y.Z., Xie L.J., Liu T.Y., Su Z.Z. (2018). Natural variation in the promoter of rice calcineurin B-like protein10 (*OsCBL10*) affects flooding tolerance during seed germination among rice subspecies. Plant J..

[34] Zhao N., Li C., Yan Y., Cao W., Song A., Wang H., Chen S., Jiang J., Chen F. (2018). Comparative transcriptome analysis of waterlogging-sensitive and waterlogging-tolerant *Chrysanthemum morifolium* cultivars under waterlogging stress and reoxygenation conditions. Int. J. Mol. Sci..

[35] Singh S., Parihar P., Singh R., Singh V.P., Prasad S.M. (2016). Heavy metal tolerance in plants: Role of transcriptomics, proteomics, metabolomics, and Ionomics. Front. Plant Sci..

[36] Zhu H., Ai H., Cao L., Sui R., Ye H., Du D., Sun J., Yao J., Chen K., Chen L. (2018). Transcriptome analysis providing novel insights for Cd-resistant tall fescue responses to Cd stress. Ecotoxicol. Environ. Saf..

[37] Grabherr M.G., Haas B.J., Yassour M., Levin J.Z., Thompson D.A., Amit I., Adiconis X., Fan L., Raychowdhury R., Zeng Q. (2011). Full-length transcriptome assembly from RNA-Seq data without a reference genome. Nat. Biotechnol..

[38] Li B., Dewey C.N. (2011). RSEM: Accurate transcript quantification from RNA-Seq data with or without a reference genome. BMC Bioinform..

[39] Anders S., Huber W. (2010). Differential expression analysis for sequence count data. Genome Biol..

[40] Storey J.D. (2003). The positive false discovery rate: A Bayesian interpretation and the q-value. Ann. Stat..

[41] Young M.D., Wakefield M.J., Smyth G.K., Oshlack A. (2010). Gene ontology analysis for RNA-seq: Accounting for selection bias. Genome Biol..

[42] Kanehisa M., Araki M., Goto S., Hattori M., Hirakawa M., Itoh M., Katayama T., Kawashima S., Okuda S., Tokimatsu T. (2007). KEGG for linking genomes to life and the environment. Nucleic Acids Res..

[43] Mao X., Cai T., Olyarchuk J.G., Wei L. (2005). Automated genome annotation and pathway identification using the KEGG Orthology (KO) as a controlled vocabulary. Bioinformatics.

[44] Dai C., Cui W., Pan J., Xie Y., Wang J., Shen W. (2017). Proteomic analysis provides insights into the molecular bases of hydrogen gas-induced cadmium resistance in *Medicago sativa*. J. Proteom..

[45] Zhang Q., Liu X., Zhang Z., Liu N., Li D., Hu L. (2019). Melatonin improved waterlogging tolerance in alfalfa (*Medicago sativa*) by reprogramming polyamine and ethylene metabolism. Front. Plant Sci..

[46] Livak K.J., Schmittgen T.D. (2001). Analysis of relative gene expression data using real-time quantitative PCR and the 2^−ΔΔ*C*t^ method. Methods.

